# *Plasmodium falciparum* specific helicase 3 is nucleocytoplasmic protein and unwinds DNA duplex in 3′ to 5′ direction

**DOI:** 10.1038/s41598-017-12927-x

**Published:** 2017-10-13

**Authors:** Manish Chauhan, Mohammed Tarique, Renu Tuteja

**Affiliations:** Parasite Biology Group, International Centre for Genetic Engineering and Biotechnology, P. O. Box 10504, Aruna Asaf Ali Marg, New Delhi, 110067 India

## Abstract

*Plasmodium falciparum* is responsible for most dangerous and prevalent form of malaria. The emergence of multi drug resistant parasite hindered the prevention of malaria burden worldwide. Helicases are omnipresent enzymes, which play important role in nucleic acid metabolism and can be used as potential targets for development of novel therapeutics. The genome wide analysis of *P*. *falciparum* 3D7 strain revealed some novel parasite specific helicases, which are not present in human host. Here we report the detailed biochemical characterization of *P*. *falciparum* parasite specific helicase 3 (PfPSH3). The characteristic ATPase and helicase activities of PfPSH3 reside in its N-terminal region (PfPSH3N) as it contains all the conserved signature motifs whereas the C-terminal does not show any detectable biochemical activity. PfPSH3N also shows DNA helicase activity in the 3′–5′ direction. The immunofluorescence microscopy results show that PSH3 is localized in nucleus as well as in cytoplasm during different stages such as trophozoite and early schizont stages of intraerythrocytic development. This report sets the foundation for further study of parasite specific helicases and will be helpful in understanding the parasite biology.

## Introduction

Malaria is a serious haematological disorder caused by the *Plasmodium* parasite transmitted to the humans by the bite of female *Anopheles* mosquito^1^. Five *Plasmodium* species such as *Plasmodium falciparum*, *Plasmodium malariae*, *Plasmodium ovale*, *Plasmodium vivax* and *Plasmodium knowlesi* are responsible for malaria of which *P*. *falciparum* poses the greatest threat to the humans due to its infection causing most severe form of the disease^2,3^. The severity of disease can be analysed through WHO report 2016 which revealed that there were 214 million new cases of malaria in December 2015 and 4,38,000 deaths were also reported^4^. Though the number of cases since 2000 to 2015 decreased by ~37% but Sub-Saharan Africa carries the major global malaria burden^4^.

Besides decline in number of cases of malaria, the parasite is becoming resistant to the modern-day combination drug therapies^5^. The parasite is already resistant to drugs used during 1970s and 1980s such as sulfadoxine-pyrimethamine (SP) and chloroquine. Due to the failure of old conventional therapy the artemisinin based combination Therapy (ACT) is being used now which includes artemisinin and other drug combinations^6^. ACT worked very well since 2000 and was widely accepted as a mainstream first line anti-malarial treatment^7^. But this drug treatment had huge toll on its effectiveness because of the emergence of artemisinin resistant parasite and the recent reports suggest that cases of artemisinin resistance are increasing at an alarming rate^4–8^.

Due to this increasing resistance towards modern day front line antimalarials there is an urgent need to develop new range of antimalarial drugs and to identify novel chemotherapeutic targets^9^. Helicases are ubiquitous and present in every organism such as bacteria, virus, yeast, plants, human, and the malaria parasite *Plasmodium* ^10,11^. Significant part of our genome encodes helicases, which proves the importance of these molecules^12^. Helicases are the molecular motors energised by the ATP (adenosine triphosphate) hydrolysis required for unwinding the duplex nucleic acid (either DNA/DNA, DNA/RNA or secondary structure in RNA)^12^. Depending on the substrate, helicases can be classified as RNA or DNA helicases, and they control various aspects of cellular homeostasis^13,14^. DNA helicases play vital role in replication^15^, repair and recombination and RNA helicases play important role in transcription, translation and RNA splicing^15,16^.

Helicases are classified into five superfamilies SF1 to SF5 depending on the presence of signature motifs^17^. Further analysis of conserved motifs of superfamily subdivided SF2 superfamily into subgroups such as DEAD box, DEAH box, and Ski2-like proteins^18,19^. DEAD box and DEAH box proteins are generally referred as DExD/H box helicases and they constitute the largest members of superfamily 2. Although all the DEAD box proteins share highly conserved core, ATP, and nucleic acid binding sites but despite structural similarities, different DEAD box proteins have unrelated functions^20–22^. Helicase core in DEAD box family is structurally conserved and contains seven to nine characteristic signature motifs^23^. Apart from the helicase core domains, most of the helicases have flanking N and C-terminal extensions, which sometimes contain additional domains for other functions^24–26^. It has been reported that these N and C terminal extensions provide specificity by helping in recruitment of these helicases to specific complexes through interaction with other proteins^27^. Helicases usually bind to single stranded region and then unwind duplex by translocating in a specific direction either 5′ to 3′ or 3′ to 5′^28–30^. The genome wide analysis of *P*. *falciparum* revealed that it contains novel helicases which are specific to parasite and their homologues are not detectable in the human host^31^. We have reported in a previous study that *P*. *falciparum* contains three parasite specific helicases (PSH)^31^. The PlasmoDb numbers of these putative helicases are PF3D7_0807100 (previous id: PF08_0111); PF3D7_0103600 (previous id: PFA0180w) and PF3D7_1202000 (previous id: PFL0100c) respectively.

In this manuscript, we report the detailed biochemical characterization of purified recombinant parasite specific helicase 3 (PfPSH3) from *P*. *falciparum* 3D7 strain. The gene was cloned in two fragments; the N-terminal fragment (PfPSH3N) of ~127 kDa contains all the characteristic helicase motifs and the C-terminal fragment (PfPSH3C) of ~30 kDa has no detectable motif. The activity analysis suggests that PfPSH3N exhibits ssDNA-dependent ATPase activity and DNA helicase activity in the 3′ to 5′ direction but PfPSH3C has no detectable enzyme activity. The polyclonal antibody generated against PfPSH3C was used to study the expression and localization of the protein in intraerythrocytic developmental stages of *P*. *falciparum* 3D7 strain using immunofluorescence assay. The results suggest that PfPSH3 protein is expressed in trophozoite and schizont stages and it is localized majorly in cytoplasm and to some extent in nucleus as well. The in silico studies suggest that PfPSH3 is a substrate for posttranslational modifications and it can interact with variety of proteins. Overall, these studies suggest that the enzymatic activities of PfPSH3 reside in its N terminal and PfPSH3 unwinds DNA duplex in 3′ to 5′ direction. The studies further suggest that in addition to nucleus during some stages of intraerythrocytic development PSH3 is also localized in the cytoplasm in *P*. *falciparum* 3D7 strain.

## Results

### *In silico* analysis of PfPSH3

**Figure 1:**
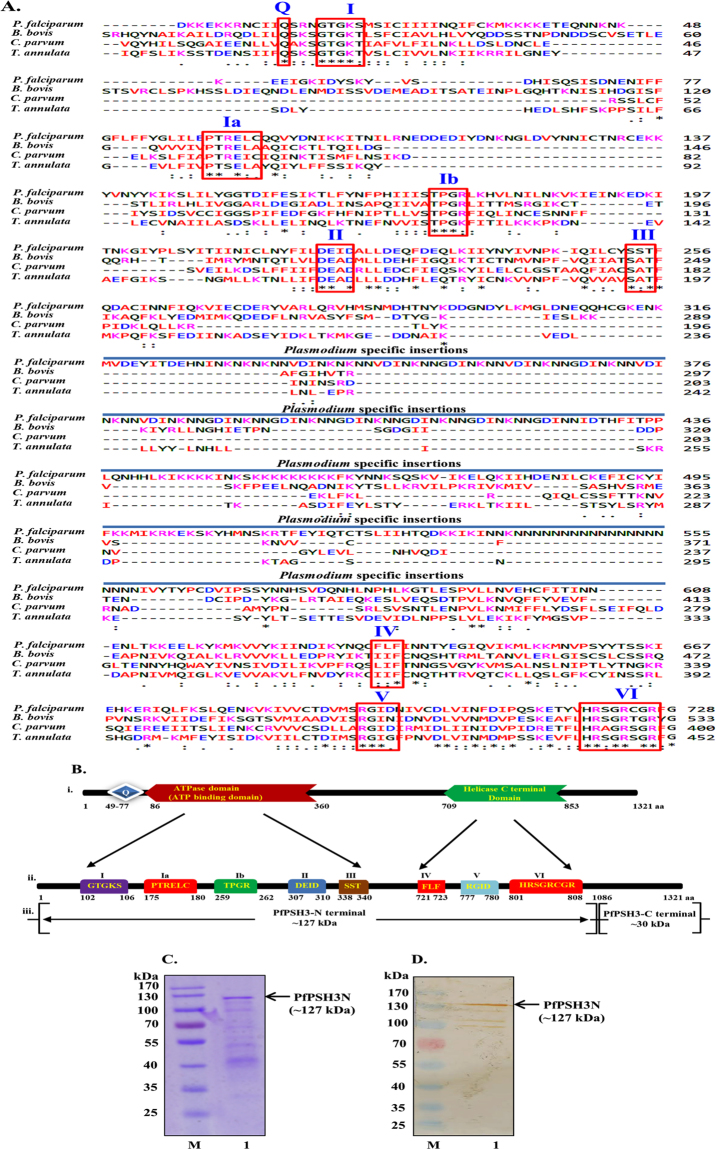
**(A**) Multiple sequence Alignment of the amino acid sequence of core region of PfPSH3 with orthologs present in Apicomplexans (*Babesia bovis*, *Cryptosporidium parvum and Theileria annulata*). The alignment was done using clustal omega program (www.ebi.ac.uk/Tools/msa/clustalo/). All signature motifs of PfPSH3 are highlighted and boxed in red colour and the name of each motif (from I to VI) is written in roman numerals; **(B)** Schematic representation of signature motifs of DEAD box family of PfPSH3. All the domains are presented with their respective amino acid positions. (**C**) Commassie blue stained gel. Lane M is molecular weight marker and lane 1 is purified PfPSH3N protein (~127 kDa); **(D)** Western Blot analysis. Lane numbers are similar to A.

PfPSH3 is a novel parasite specific helicase with PlasmoDb number PF3D7_0807100 (previous id PF08_0111)^31^. Orthologs of PfPSH3 were identified using online available database OrthoMCL^32^, it uses Markov cluster algorithm to identify groups paralogs and orthologs. The results revealed that the ortholog group OG5_147475 is only present in alveolata apicomplexans group i.e. *Babesia bovis*, *Cryptosporidium parvum*, and *Theileria annulata*. The core amino acid sequence of PfPSH3 containing all the seven conserved signature motifs was used for multiple sequence alignment with orthologs present in other organisms using Clustal omega^33,34^. This analysis revealed that long insertions are present in between conserved signature motifs in all the Plasmodium species including PfPSH3 but are absent in other orthologs (Fig. 1A; Supplementary Fig. 1). It is noteworthy that PfPSH3 contains serine in place of threonine in motif I, isoleucine in place of alanine in motif II and serine in place of alanine in motif III (Fig. 1A). In other Plasmodium species, valine is present in place of alanine in motif II (Supplementary Fig. 1). The detailed domain organization of PfPSH3 using Prosite (www.http://prosite.expasy.org/prosite.html) provided the predicted position of both ATPase domain (from amino acid 86–360) and helicase C terminal domain (from amino acid 709–853) (Fig. 1Bi). All the signature motifs of PfPSH3 such as Q, I, Ia, II, III, IV, V and VI present within both ATP binding and helicase C-terminal domains with their respective amino acid positions are also shown^35^ (Fig. 1Bii).

### Structural modelling of PfPSH3

In order to study the structural homology of PfPSH3, its structure was predicted using the N-terminal (1–1086) amino acid sequence. The sequence was submitted to the Swissmodel homology modelling server (http://swissmodel.expasy.org/)^36,37^. Swissmodel server searched for the template that has the most coverage and identity score for making homology model and used 4tyw.1.A (Mss116, mitochondrial ATP dependent RNA helicase, *Saccharomyces cerevisiae*) as a template. UCSF Chimera software^38^ was used for presenting the images of pdb files of template and model (Supplementary Fig. 2Ai–iii). The structural modelling results suggest that PfPSH3N is structurally similar to a mitochondrial helicase of *S*. *cerevis* *iae* ^39^. For further structural analysis of predicted model, PdbSUM suite Procheck (http://www.ebi.ac.uk/thornton-srv/databases/cgi-in/pdbsum/GetPage.pl?pdbcode=index.html) was used^40^. The Ramachandran plot obtained for the modelled structure further revealed that residues in most favoured regions and additional allowed regions are 66% and 25% respectively, while the residues in generously allowed regions and disallowed regions are only 5% and 3%, respectively (Supplementary Fig. 2B).

### Purification of PfPSH3N and PfPSH3C proteins

PfPSH3 is present on chromosome number 3 of *P*. *falciparum* 3D7 from locus 372,902 to 376,876 (3966 base pair) and designated as protein coding gene with amino acid sequence length of 1321 amino acid. The calculated molecular weight is ~157 kDa and the domain analysis revealed that two functional domains i.e. ATP binding domain and helicase domain, starting from 68 to 814 amino acids are present in the N-terminal of the protein and the flanking C-terminal region contains no detectable domain (Fig. 1Bii). The amplification of full-length gene using appropriate set of primers was unsuccessful on repeated trials. Therefore, the primers were designed to amplify the N-terminal region (1–3260 base pair; coding for ~127 kDa protein) carrying both the functional domains and the C-terminal region of 726 bp (3240–3966 base pair; coding for ~30 kDa protein) separately (Fig. 1Biii). The genomic DNA of *P*. *falciparum* was used as template and the amplified fragments were subsequently cloned in cloning vector pJET 1.2 (Supplementary Fig. 3A).

To overexpress the PfPSH3N and PfPSH3C proteins in *E*. *coli* both the N and C terminal fragments were sub-cloned into pET28a + expression vector and transformed in BL21 codon plus expression cells. These proteins were expressed at 16 °C and subsequently purified using Ni-NTA affinity chromatography. The purified fractions of PfPSH3N and PfPSH3C were confirmed by western blot analysis using the monoclonal anti-his antibody, which confirmed the presence of his-tagged recombinant PfPSH3N (Fig. 1C and D, lane 1) and PfPSH3C (Supplementary Fig. 3B and C, lane 1). The purified fractions of PfPSH3N and PfPSH3C were used for all the enzyme assays and PfPSH3C was also used for the generation of polyclonal antibodies in rabbit.

### ATPase activity assay of PfPSH3N and PfPSH3C

**Figure 2:**
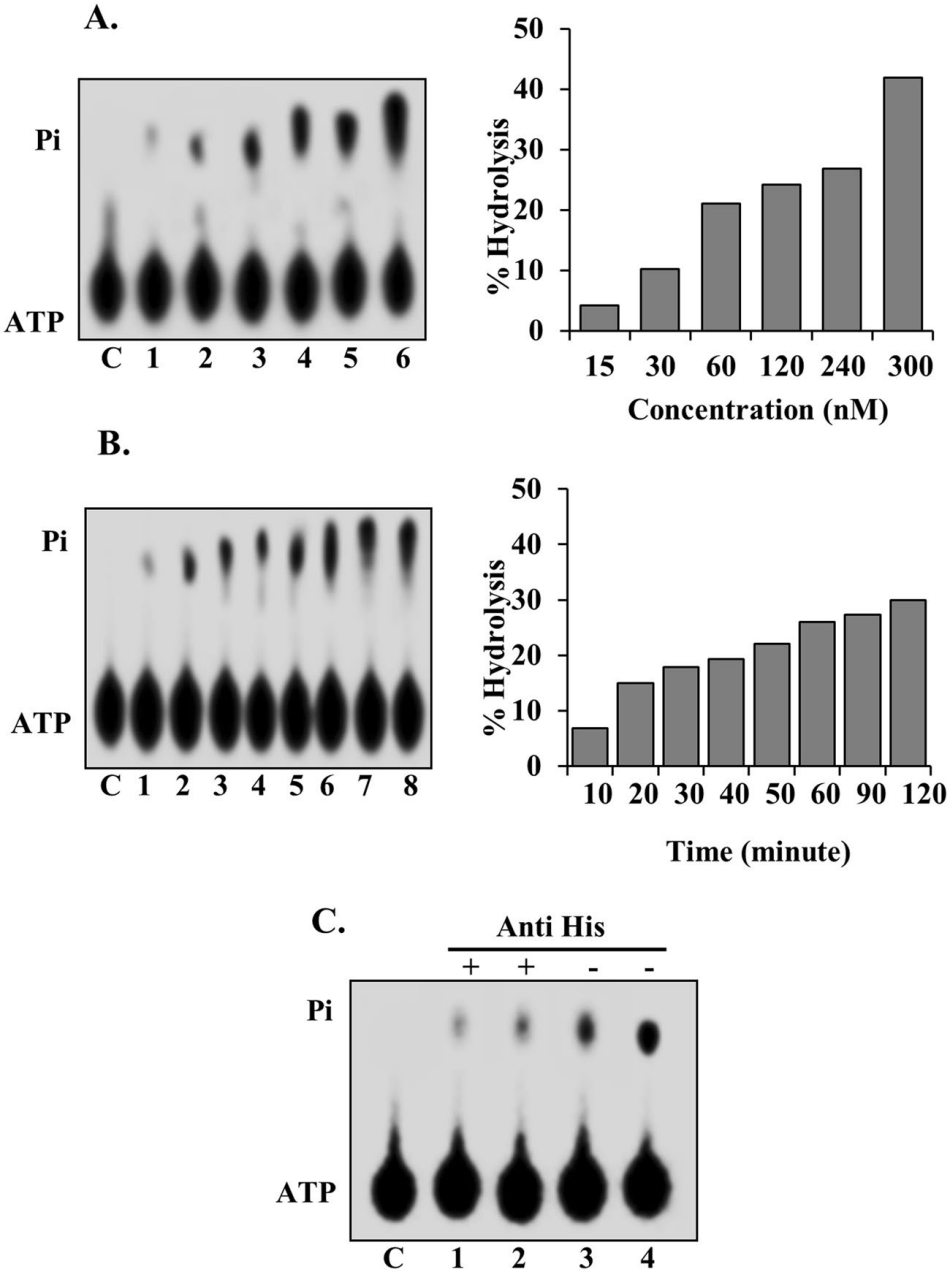
ATPase activity analysis of PfPSH3N. (**A)** ATPase activity in the presence of ssM13 DNA with increasing concentration (15 nM to 300 nM) of PfPSH3N protein (lanes 1–6), lane C is negative control reaction without protein; (**B)** Time dependent ATPase activity of PfPSH3N (lanes 1–8) and lane C represents negative control. The data are presented in graphical form at the right-side panel of A and B; (**C)** Immunodepletion assay of PfPSH3N ATPase activity. Lanes 1–2, represent reactions with increasing concentrations of supernatant of PfPSH3N pre-treated with monoclonal anti-his antibody and lanes 3 and 4 show reactions without anti-his antibody.

To analyse the ATPase activity of PfPSH3N, radiolabelled (γ^32^P) ATP was used and release of γ^32^P labelled phosphate (Pi) was measured as described in methods section. Varying concentrations of PfPSH3N (15 nM to 300 nM) were used to observe the release of Pi (Fig. 2A). The results show that PfPSH3N exhibits concentration dependent ATPase activity (Fig. 2A, lanes 1 to 6). To study the time-dependence of ATPase activity, 240 nM of PfPSH3N protein was used and the results revealed that ATPase activity of PfPSH3N is time dependent. The ATPase activity was detectable after 20 minutes of incubation and a maximum of 32% hydrolysis occurred after 60 minutes of incubation (Fig. 2B, lanes 1–8). These observed results suggest that PfPSH3N possesses active concentration and time-dependent ATPase activity. PfPSH3C did not show any detectable ATPase activity due to lack of conserved domain in this fragment of the protein (Supplementary Fig. 4, lanes 1–6).

### Immunodepletion assay of ATPase activity

Immunodepletion was performed to determine whether the ATPase activity exhibited by the protein is specific to PfPSH3N. The anti-his antibody was used for immunodepletion assay because recombinant PfPSH3N protein contains 6X his tag. To check specificity of the ATPase activity, PfPSH3N was incubated with or without anti-his antibody separately and the supernatants were used for the assay. The results indicate that ATPase activity is specific to PfPSH3N because the activity was very less in supernatant of the reaction mixture treated with anti-his antibody as compared to the supernatant of reaction mixture without anti-his antibody (Fig. 2C, lanes 1–4).

### Helicase activity assay of PfPSH3N

**Figure 3:**
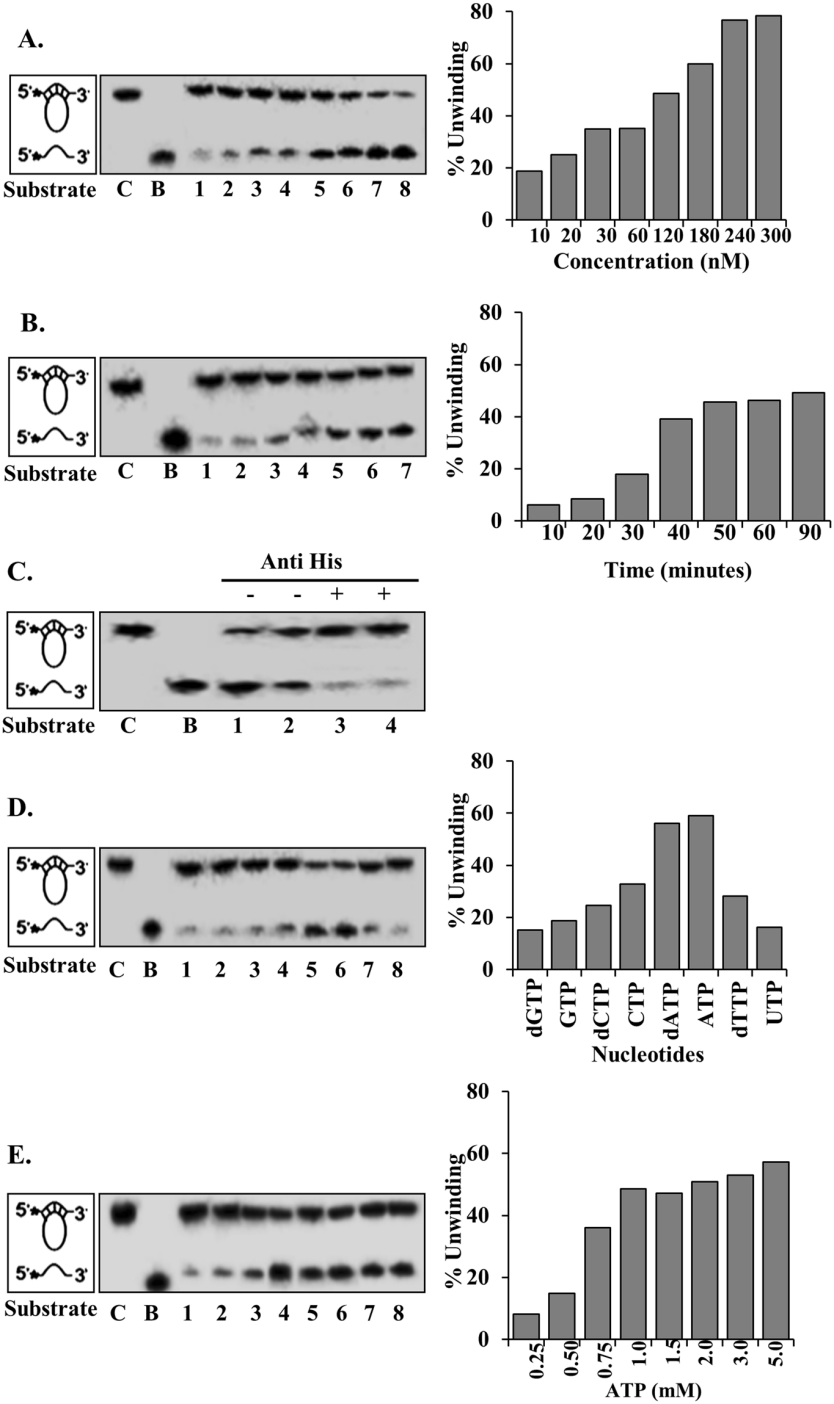
Helicase activity assay of PfPSH3N. **(A)** Lanes 1–8 are reactions with increasing concentration (10 nM to 300 nM) of PfPSH3N protein; (**B)** Lanes 1–8, show time dependant helicase activity with fixed concentration (180 nM) of PfPSH3N protein; (**C)** Immunodepletion of helicase activity. Lanes 1 and 2 are reactions with increasing concentration of PfPSH3N without anti-his antibody and lane 3 and 4 are reactions with increasing concentrations of supernatant of PfPSH3N pre-treated with monoclonal anti-his antibody. (**D)** Helicase activity of PfPSH3N in presence of different nucleotides/deoxynucleotides such as GTP, dGTP, dCTP, CTP, dATP, ATP, dTTP and UTP (lanes 1–8); (**E)** Lanes 1–8, helicase activity in presence of different concentrations of ATP (0.5 to 5 mM). In (**A**–**E)**, lane C is control reaction without enzyme and B is boiled substrate. The data of (**A**,**B**,**D** and **E**) are shown as bar diagram on the right side.

Helicases exhibit the ability to unwind the nucleic acid duplex by harnessing energy derived from ATP hydrolysis. Purified PfPSH3N was used to perform DNA helicase assay with partially duplex DNA substrate as described in methods section. The reaction mixture containing substrate and PfPSH3N protein was incubated for 60 minutes at 37 °C. The radiolabelled oligonucleotide separated from the duplex DNA substrate due to unwinding activity of PfPSH3N. The helicase activity was measured using varying concentration (10 nM to 300 nM) of PfPSH3N protein and up to 78% percent unwinding of duplex DNA substrate was observed (Fig. 3A, lanes 1–8). To determine the time-dependent helicase activity of PfPSH3N, the assay performed with fixed concentration of protein (180 nM) was incubated for different times and the results show that the helicase activity is time-dependent (Fig. 3B, lanes 1–7).

### Immunodepletion assay of helicase activity

Immunodepletion was also done to determine whether the helicase activity is specific to PfPSH3N protein. The purified protein was incubated with or without anti-his antibody separately and the supernatants were used for the assay. The results indicate that helicase activity is also specific to PfPSH3N because the activity was very low in supernatant of the reaction mixture treated with anti-his antibody (Fig. 3C, lanes 3 and 4) as compared to the supernatant of reaction mixture without anti-his antibody (Fig. 3C, lanes 1 and 2).

### Nucleotide dependency assay for helicase activity

To determine which NTP/dNTP (nucleotide triphosphate/deoxynucleotide triphosphate) PfPSH3N prefers, all of these such as dGTP, GTP, dCTP, CTP, dATP, ATP, dTTP and UTP were used for the helicase assay. The results indicate that PfPSH3N (180 nM) showed maximum helicase activity in the presence of dATP (58%) and ATP (60%) (Fig. 3D, lanes 5 and 6). Whereas with CTP it showed moderate activity of 35% (Fig. 3D, lane 4) and with dGTP, GTP, dCTP, dTTP and UTP it exhibited considerably lower activity (Fig. 3D, lanes 1–3 and lanes 7 and 8, respectively). These results suggest that for unwinding duplex DNA substrate, PfPSH3N requires ATP or dATP. The optimum concentration of ATP required to obtain maximum unwinding was assayed using ATP concentration ranging from 0.25 to 5.0 mM. Maximum unwinding of 55% was observed in the range of 1 to 2 mM ATP concentrations (Fig. 3E, lanes 4–6).

### Direction specificity of PfPSH3N

**Figure 4:**
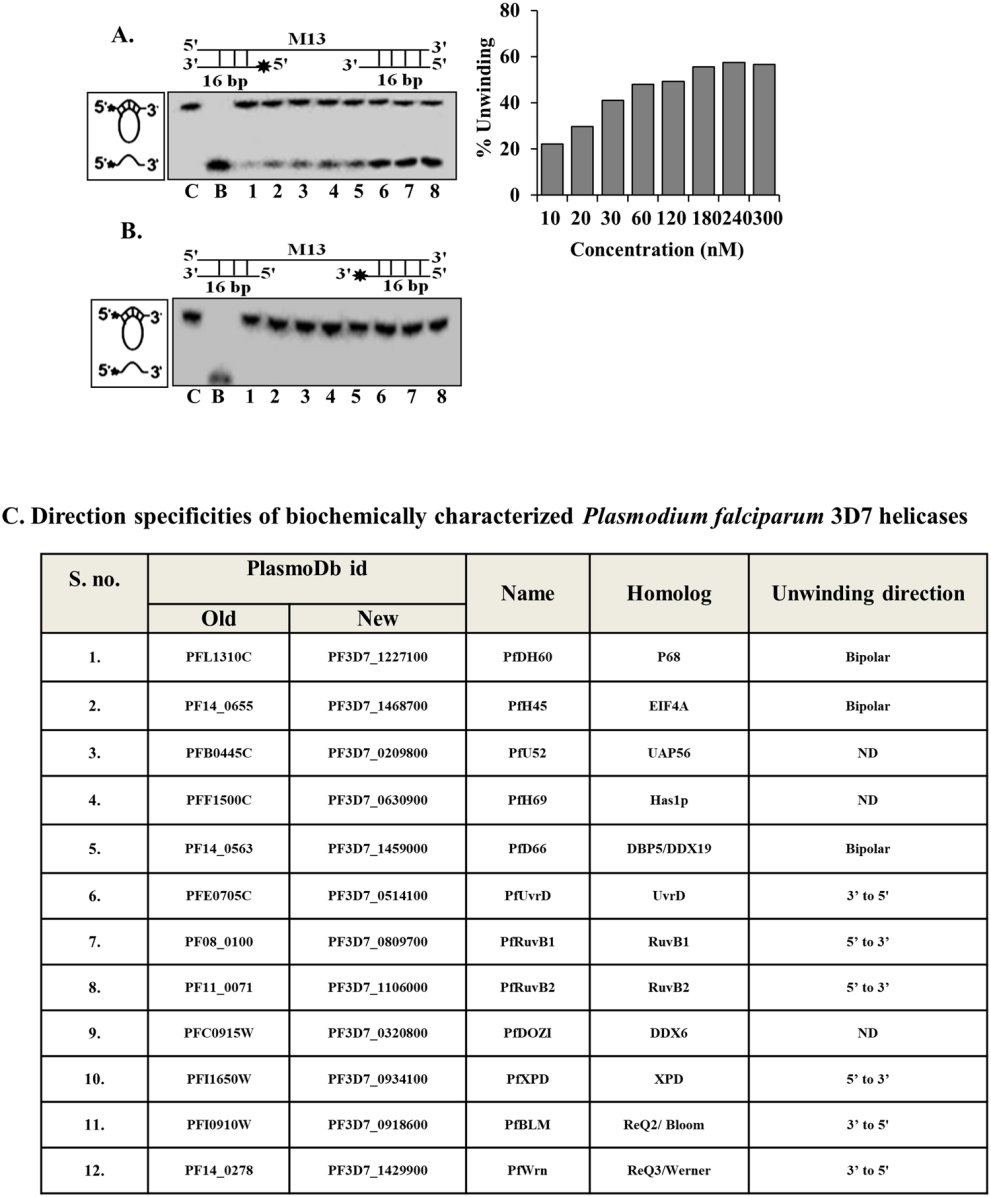
Direction specific helicase activity of PfPSH3N. (**A)** Helicase activity of PfPSH3N with the 3′ to 5′ direction specific substrate. Lanes 1–8 are reactions with increasing concentration (10 nM to 300 nM) of PfPSH3N and graphical representation of quantitative data are shown on right side; (**B)** Helicase activity of PfPSH3N with the 5′ to 3′ direction specific substrate. Lanes 1–8 are reactions with increasing concentration of PfPSH3N. (**C**) Direction specificities of biochemically characterized *P*. *falciparum* 3D7 helicases. ND is not determined.

DNA is double stranded molecule and to unwind the duplex substrate, the helicase must have some direction specificity. This direction-specificity is determined by the way protein is loaded and then moves in the specific direction i.e. either 3′ to 5′ or 5′ to 3′^41^. The unwinding directionality of PfPSH3N was analysed by using two different direction-specific substrates prepared using the method described in methods section. The helicase activity of PfPSH3N was determined using varying concentrations (10 nM to 300 nM) of protein. The results indicate that PfPSH3N shows up to 58% unwinding with the 3′ to 5′ direction-specific substrate (Fig. 4A, lane 7). However, PfPSH3N was unable to show the activity with 5′ to 3′ direction-specific substrate (Fig. 4B, lanes 1–8). These observations suggest that the direction of translocation of PfPSH3N is 3′ to 5′.

### *In vivo* expression and localization of PSH3

**Figure 5:**
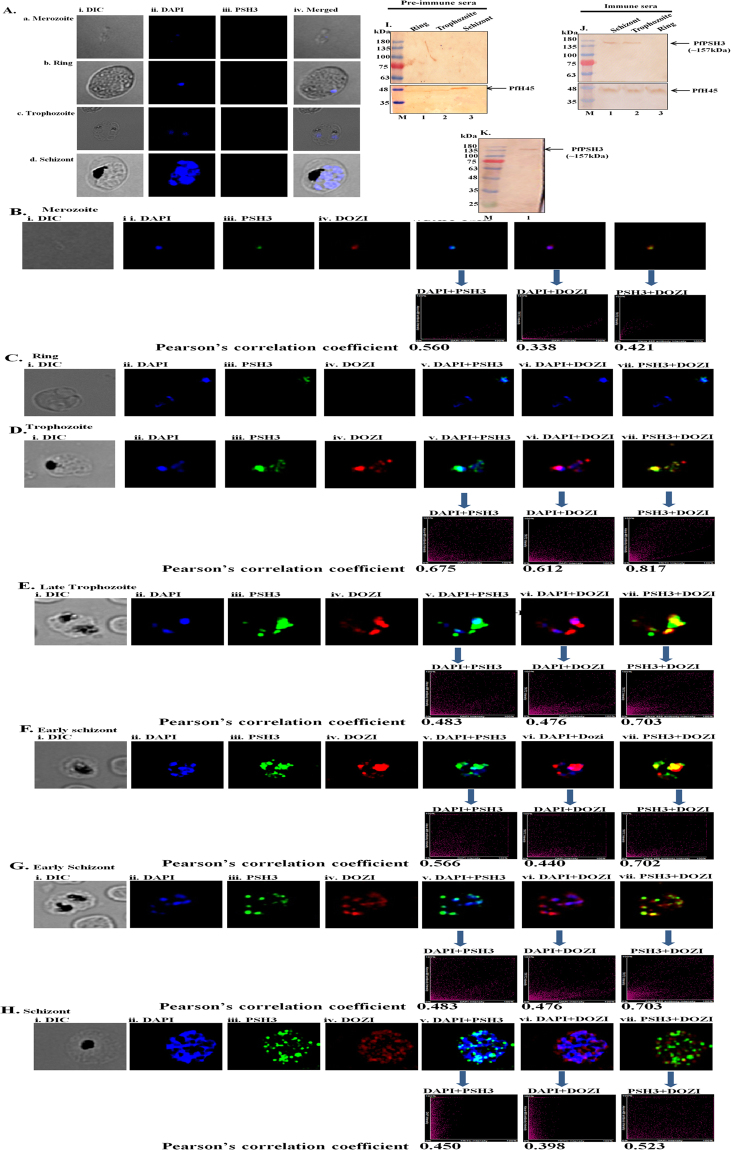
Localization of PSH3 in different intra-erythrocytic stages of *P*. *falciparum*. The cells were fixed and stained with pre-immune sera or anti-PfPSH3C antisera and anti-PfDOZI antisera followed by Alexa fluor 488 and Alexa fluor 594-conjugated secondary antibodies and then counterstained with DAPI. In each panel, single confocal image of each stage is shown. **(A)** Staining with pre-immune sera (a) merozoite stage, (b) Ring stage, (c) Trophozoite stage, (d) Schizont stage, (i) phase contrast (DIC) image; (ii) image of cell stained with DAPI (blue); (iii) immunofluorescent stained cell; (iv) All merged. **(B**–**H)** Staining with anti-PSH3 and anti-DOZI sera (**B**) merozoite stage, (**C**) Ring stage, (**D**) Trophozoite stage, (**E**) Late Trophozoite stage (**F**,**G**) Early Schizont stage, (**H**) Schizont stage (i) phase contrast (DIC) image; (ii) image of cell stained with DAPI (blue); (iii) immunofluorescent stained cell (PSH3); (iv) immunofluorescent stained cell (DOZI); (v) Merged image of panel ii and iii; (vi) Merged image of panel ii and iv (vii) Merged image of panel iii and iv. Pearson’s correlation coefficients of merged images are written. **(I)** Western blot analysis of different intraerythrocytic developmental stages of *P*. *falciparum* 3D7 strain using pre-immune serum. Lanes 1–3 are lysates from ring, trophozoite and schizonts, stages. **(J)** Western blot analysis of different intraerythrocytic developmental stages of *P*. *falciparum* 3D7 strain using anti-PSH3 serum. Lanes 1–3 are lysates from schizonts, trophozoite and ring stages, PfH45 was used as a loading control in I and J. **(K)** Western blot analysis for PSH3 using parasite lysate prepared from mixed stage parasite culture of *P*. *falciparum* 3D7 strain using anti-PSH3 serum. Lane M is protein marker and lane 1 is parasite lysate. Lane M in I, J and K is prestained protein molecular weight marker. The arrows show the PfPSH3 band.

Different intraerythrocytic asexual developmental stages of *P*. *falciparum* 3D7 strain were used to study localization of PSH3. DAPI stain was used to locate nucleus and for PSH3 staining, anti-PfPSH3C antibody along with secondary antibody conjugated to Alexa Fluor 488 dye (green) was used. The pre-immune sera did not show any staining in different intraerythrocytic developmental stages (Fig. 5A,a–d; Supplementary Fig. 5A–D). To check whether PSH3 is also localized in the cytoplasm, the staining with antibodies against cytoplasmic helicase PfDOZI was also performed^42^. Secondary antibodies conjugated to alexa fluor 594 dye (red) were used to determine the colocalization (Fig. 5B–H). The results suggest that PSH3 was not detectable in ring stages of development of the parasite (Fig. 5Ci–vii). The results further suggest that expression of PSH3 initiates from trophozoite stage of intraerythrocytic development and it is localized mostly in cytoplasm and to some extent in nucleus also at trophozoite, early schizont and late schizont developmental stages. PSH3 colocalizes with cytoplasmic helicase DOZI in the trophozoite and early schizont stages and the Pearson’s correlation coefficient ranges from 0.8 to 0.7 (Fig. 5 panel vii of D-G). The preimmune serum and purified antibodies were also used to detect the level of expression of PSH3 in the *P*. *falciparum* 3D7 parasite lysate. There was no detectable protein in the parasite lysate of different intraerythrocytic developmental stages (ring, trophozoite and schizont) when the blot was probed with preimmune serum (Fig. 5I, lanes 1–3). For stage specific expression, western blots were prepared by using parasite lysate of different intraerythrocytic developmental stages (ring, trophozoite and schizont). PfPSH3 antibodies recognized single band in schizont and trophozoite stages of the parasite (Fig. 5J, lanes 1 and 2) but there was no expression observed in ring stages of the parasite (Fig. 5J, lane 3). PfH45 was used as a loading control suggesting that equal amount of protein is loaded in each lane. The purified IgG recognized only a single protein of right size (~157 kDa) in the lysate prepared from a mixed intraerythrocytic developmental stages (ring, trophozoite and schizont) of malaria parasite-infected RBCs **(**Fig. 5K, lane 1).

### Prediction of post translational modifications and interacting partners of PfPSH3

Post translational modifications (PTMs) play crucial role in diversifying the functional ability and complexity of proteins^43^. PTMs are covalent addition and or removal of functional groups to specific amino acid residues in a protein^44^. Major PTMs involved in biological system are phosphorylation, glycosylation, ubiquitination, methylation and acetylation. Phosphorylation prediction of PfPSH3 was done using NetPhos 3.1 (http://www.cbs.dtu.dk/services/NetPhos/)^45^ and the results suggest that there are various phosphorylation sites present in PfPSH3 (Supplementary Table 1A). These include phosphorylation by PKC at various places including threonine at position 103 in motif I (Supplementary Fig. 6). Similarly, methylation prediction was detected using PSSME (Prediction of species-specific Methylation sites) (http://bioinfo.ncu.edu.cn/PSSMe.aspx)^46^ (Supplementary Table 1B) and lysine specific methylation was observed at various positions (Supplementary Fig. 6). Acetylation was also predicted using GPS-PAIL (prediction of acetylation of internal lysine) (http://pail.biocuckoo.org/)^47^ (Supplementary Table 1C) (Supplementary Fig. 6). It was observed at lysine at position 105 in motif I.

**Figure 6:**
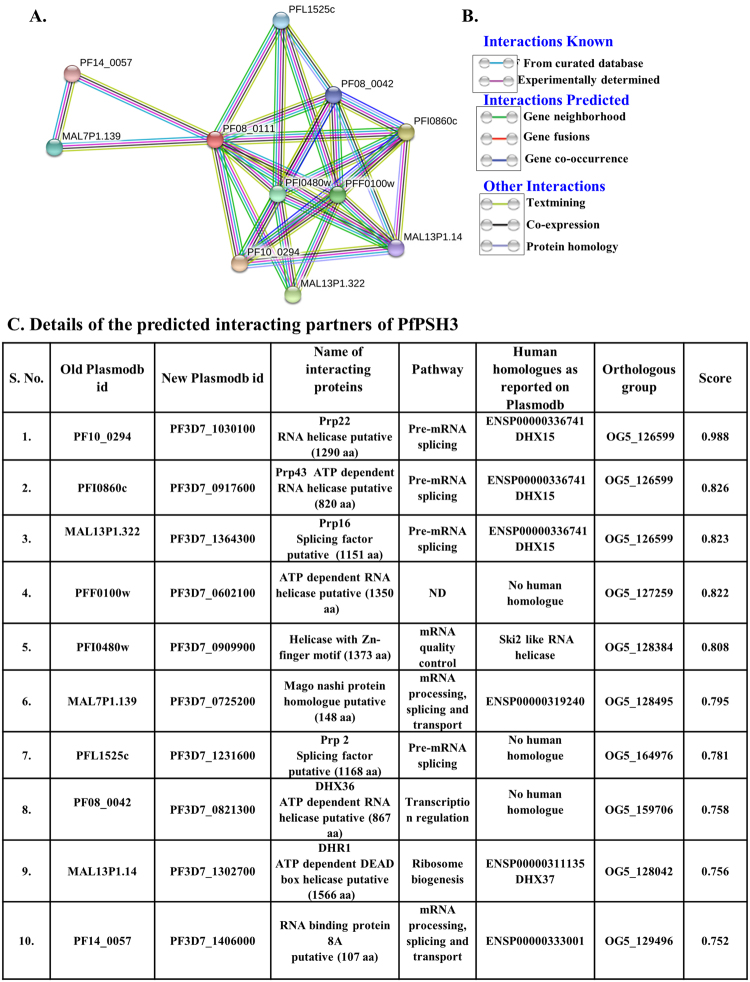
Protein-protein interaction prediction. **(A)** The results show the proteins interacting with PfPSH3. The Plasmodb numbers of all are shown in the model. The cutoff score of 0.7 was used and only top ten interactors are shown. (**B**) Explanation of interactions shown in A. (**C**) Details of the predicted interacting partners of PfPSH3.

The prediction of interacting partners of PfPSH3 was done using the STRING-DB Version 10.5 (http://string-db.org/)^48,49^. This analysis predicted the interacting partners, which have probable roles in various cellular pathways such as splicing, gene regulation and ribosome biogenesis. The partners predicted are five ATP-dependent DEAD-box putative RNA helicases, pre-mRNA splicing factor RNA helicase, putative helicase with Zn finger motif, putative splicing factor and putative RNA binding protein (Fig. 6B). The pathway in which these proteins are involved and their functional roles are also described (Fig. 6B).

## Discussion

Helicases are vital class of enzymes necessary for various nucleic acid metabolic pathways and studying these enzymes will help to understand the malaria parasite biology. *P*. *falciparum* contains variety of helicases as reported in earlier studies^50^. The characterization of few *P*. *falciparum* helicases has been reported previously^30,51–54^ but still the biochemical characterization of other helicases is required for better understanding of the role of these vital enzymes in the parasite. Here we report the biochemical characterization of a novel helicase that is specific to the malaria parasite. The in-silico analysis revealed that it has no homologs present in human host. The orthologs present are with very less identity percentage i.e. 34.2% that too only due to identity between orthologs of *Plasmodium* species. The bioinformatics analysis confirmed the presence of all seven-conserved signature motifs required for helicase and ATPase activity but in motif II, PfPSH3 contains DEID in place of usual conserved sequence DEAD. Due to the large size of the gene, it was amplified in two fragments and two proteins were purified and characterized. The in-silico modelling of PfPSH3N suggested that it is structurally similar to mitochondrial ATP dependent RNA helicase MSS116 of *S*. *cerevisiae* ^39^. The biochemical characterization further shows that purified PfPSH3N is ssDNA-dependent ATPase and 3′–5′ direction specific DNA helicase. PfPSH3N contains all the conserved motifs and is catalytically active whereas no detectable motifs are present in PfPSH3C and it is catalytically inactive. These observations suggest that PfPSH3 retains its helicase and ATPase activity despite lacking the non-conserved C-terminus of ~30 kDa. In the case of PfWrn also the enzymatic activities are present in the ~62 kDa N terminal region of the protein because no motifs were detectable in the ~100 kDa C terminal of PfWrn^55^. But in case of PfH69 it has been reported previously that the N terminal of the protein is required for its enzymatic activities even though no detectable motifs are present in its N terminal region^56^. Most likely, the removal of N terminal region of PfH69 destabilizes the protein and affects its enzymatic activities. On the other hand, no such destabilization was observed by truncation of the C terminal region of PfPSH3.

Helicase activity of PfPSH3N is nucleotide dependent and it showed maximum unwinding activity with ATP as compared to other nucleotide triphosphates. PfPSH3N prefers ATP to other nucleotide triphosphates as substrate for duplex unwinding. However, there are reports where helicases also use other nucleotide triphosphates with similar preferences. It has been reported that PfWrn also can utilize GTP to similar extent^55^. On the other hand, PfUvrDN can utilize all the nucleotide triphosphates for its unwinding activity^57^. For unwinding of duplex nucleic acids, the helicases generally translocate unidirectionally but few helicases also exhibit bipolar activity. The direction of unwinding of the biochemically characterized helicases from *P*. *falciparum* 3D7 has been summarized (Fig. 4C). Some of the helicases such as PfDH60, PfH45 and PfD66 exhibit bipolar unwinding activity (Fig. 4C). Similar to most of the other biochemically characterized helicases PfPSH3N also exhibits 3′–5′ direction specific DNA unwinding activity.

The localization studies revealed that PSH3 is present in the nucleus in early intraerythrocytic developmental stages and later it is also present in the cytoplasm. The colocalization studies with cytoplasmic helicase PfDOZI confirmed the presence of PSH3 in the cytoplasm^42^. Although PfWrn is localized in nucleus as well as in cytoplasm, it exhibits DNA helicase activity similar to other Wrn protein homologues^55,58^. PfPSH3 also shows similar pattern of nucleocytoplasmic localization and contains DNA helicase activity. According to the RMgmDB (Rodent Malaria genetically modified parasites data base) gene disruption studies have been done on the ortholog of PfPSH3 (PF3D7_0807100) which is PBANKA_1223500. It has been reported that the gene is likely essential for asexual blood stages growth/multiplication as determined by barcode PCR in a large pool of gene-deletion mutants (http://www.pberghei.eu/index.php?rmgm=3290&hl=PF3D7_0807100) (Supplementary Fig. 7).

Post translational modifications are responsible for the diversity of proteins and are very important for a specific biological function or an event^43^. Prediction of PTMs in PfPSH3 revealed that it contains various sites for modification, which may be important for its functional activity or interaction with the other proteins involved in same or different pathways at specific time and location. Different predicted PTMs attributed to PfPSH3 are phosphorylation, acetylation and methylation. Motif I (GTGKS) of PfPSH3 present in the ATPase domain contains a predicted phosphorylation site at threonine residue and acetylation site at lysine reside suggesting that these modifications might play role in its functional regulation. Various lysine methylation sites were also predicted for PfPSH3, which may be playing crucial role in regulation of PfPSH3. The in-silico protein-protein interaction studies suggest that various proteins interact with PfPSH3 such as splicing factors and ATP dependent RNA helicases. The interaction predictions hint about the presence of PfPSH3 in different cellular compartments of the parasite. The nucleocytoplasmic presence of PfPSH3 in intraerythrocytic developmental stages suggests that PfPSH3 might be interacting with proteins involved in variety of nucleic acid metabolic pathways.

To the best of our knowledge, this is the first report of detailed characterization of parasite specific helicase 3. The results reported in this study will improve our understanding of the parasite specific helicases and their function in the parasite. This study also paves way for further study of the parasite specific helicases and will help to delineate the pathways in which this helicase might be involved in the parasite.

## Methods

### In-silico analysis

PlasmoDb (release 27) database was used to retrieve sequence of PfPSH3 (PF3D7_0807100). Schematic diagram for PfPSH3 was generated using Prosite (http://prosite.expasy.org/prosite.html)^34^. Sequences of orthologues of PfPSH3 were obtained from NCBI and multiple sequence alignments were done using Clustal Omega^33^ (http://www.ebi.ac.uk/Tools/msa/clustalo/).

### Parasite blood stage culture

*P*. *falciparum* 3D7 strain was cultured in human erythrocytes (4% haematocrit) in RPMI media (Invitrogen Corporation, USA), 50 mg/L hypoxanthine (Sigma Aldrich Co., USA), 0.5 g/L Albumax I (Gibco, Thermofisher Scientific Inc., USA) and 2 g/L sodium bicarbonate (Sigma Aldrich Co., USA) supplemented with O^+^ human serum using standard protocol^59^. The serum was obtained from Rotary Blood Bank, New Delhi, India. The cultures were synchronized with 5% sorbitol for at least two successive cycles and harvested using saponin treatment^60^.

### Expression and purification of recombinant proteins

PfPSH3N-pET28a + clone was transformed into *E*. *coli* strain BL21 codon plus cells. After addition of 1 mM IPTG, the secondary culture was further allowed to grow at 16 °C for 12 hours. Lysis buffer of pH 7.5 (50 mM Tris–HCl, 250 mM NaCl, 0.05% Tween 20, 0.1% Triton 100 and the protease inhibitor cocktail from Sigma, St. Louis, MO, USA) was used and the lysate was centrifuged. The protein in supernatant was subjected to binding with Ni-NTA (Qiagen, GmbH, Germany) equilibrated in binding buffer (50 mM Tris–HCl pH 7.5, 250 mM NaCl, 10 mM imidazole) and protease inhibitor cocktail for 1 hr at 4 °C. The recombinant his-tagged protein was eluted with imidazole (50–200 mM) in chilled elution buffer (50 mM Tris–HCl pH 7.5, 250 mM NaCl, 10% (v/v) glycerol and protease inhibitor cocktail). SDS-PAGE coupled western blot analysis was performed for checking purity of PfPSH3N. For western blot analysis anti His-tagged antibodies conjugated with horse radish peroxidase (Sigma, St. Louis, MO, USA) were used for detection. The purification of PfPSH3C was done using similar method.

### Ethics Statement

The animal studies described in this study were approved by the ICGEB Institutional Animal Ethics Committee (IAEC Reference No. 53–3). ICGEB is licensed to conduct animal studies for research purposes under the registration number 18/1999/CPCSEA (dated 10/1/99). This is to further state that all experiments were performed in accordance with relevant guidelines and regulations.

### Polyclonal antibody generation against PfPSH3C protein

For the generation of polyclonal sera in rabbit, purified PfPSH3C protein was used. Equal volume of PfPSH3C protein and complete Freunds adjuvants were mixed properly for preparing emulsion. The rabbit was injected using this mixture and the booster dose was given by using mixture of pure PfPSH3C protein with incomplete Freunds adjuvant.

### ATPase assay

The ATPase activity was assayed by measuring the released Pi from [γ-^32^P] ATP. The purified PfPSH3N protein was mixed with buffer (20 mM Tris–HCl, pH 8.0, 8 mM DTT, 1.0 mM MgCl_2_, 20 mM KCl and 16 μg/ml BSA) and a mixture of [γ-^32^P] ATP (~17 nM) and 1 mM cold ATP and it was incubated at 37 °C for 1 hr. For checking DNA-dependant ATPase activity 50 ng of M13 mp19 ssDNA was added in the above reaction and negative control was without protein. The reaction was quenched on ice after 1 hr of incubation and then 1 μl of reaction mix was spotted onto thin layer chromatography (TLC) plate (Sigma). TLC buffer (0.5 MLiCl and 1 M formic acid) was used for separating hydrolysed Pi using TLC and the plate was air dried and then scanned on phosphoimager. All the quantitation was done using Image j software^61^.

### Immunodepletion assay

In order to confirm that the enzyme activities are specific to PfPSH3N protein, monoclonal anti-His antibody was mixed with purified recombinant PfPSH3N protein and incubated at 0 °C for 2 hrs on ice. Antigen–antibody complex was removed by the addition of equilibrated protein A sepharose beads and the supernatant after centrifugation was directly used for the ATPase activity in the same way as described above.

### Preparation of DNA helicase substrate and direction-specific substrates

The helicase activity of PfPSH3N was checked by the standard strand displacement assay. The partially duplex substrate consisted of a ^32^P-labelled 47-mer DNA oligodeoxynucleotide annealed to M13mp19 ssDNA. This oligodeoxynucleotide 5′-(T)_15_GTTTTCCCAGTCACGAC(T)_15_–3′ contains 15 base-pairs of non-complementary region (T)_15_ at both the 5′ and 3′ ends. It was labelled at 5′-end with T4 polynucleotide kinase (PNK) (5U) (New England Biolabs) and 1.85 MBq of [γ-^32^P] ATP (specific activity 222 TBq/mmol) at 37 °C for 1 hr and then annealed using standard annealing buffer (20 mM Tris-HCl, pH 7.5, 10 mM MgCl_2_, 100 mM NaCl, 1 mM DTT) with 0.5 μg of M13mp19 ssDNA. Sepharose 4B column (Pharmacia, Sweden) was used for removing non-hybridized oligodeoxynucleotide. The eluted fractions of substrate were checked on a nondenaturing 12% PAGE by electrophoresis and then purified fractions were used for helicase assay.

To check the direction of unwinding activity of PfPSH3N, the direction specific substrates were prepared. The substrate consisting of long linear M13 mp19 ssDNA with short duplex ends for 3′ to 5′ unwinding was prepared by first 5′-end labelling of 32-mer oligodeoxynucleotide (5′-TTCGAGCTCGGTACCCGGGGATCCTCTAGAGT-3′) and then annealing as described above. This derived duplex substrate was digested with SmaI and purified by gel filtration through sepharose 4B. For constructing the 5′ to 3′ direction-specific substrate, the oligodeoxynucleotide 32-mer was first annealed to M13mp19 ssDNA and then labelled at 3′-OH end in appropriate buffer with 50 μCurie (α-^32^P) dCTP and 5 units of DNA polymerase I (large fragment) at 23 °C for 20 min. The incubation was continued for additional 30 min at 23 °C after increasing the dCTP concentration to 50 mM using unlabelled dCTP. The annealed substrate was digested with SmaI and purified as described above.

### DNA helicase assay

Each reaction mixture (10 µl) contained purified protein, helicase buffer (20 mM Tris–HCl, 8 mM DTT, 1 mM MgCl_2_, 1 mM ATP, 10 mM KCl, 4% (w/v) sucrose, 80 µg/ml BSA), ^32^P-labelled helicase substrate (~1000 cpm) and incubated at 37 °C for 60 min. The reaction was stopped by the addition of helicase dye (0.3% SDS, 10 mM EDTA, 5% glycerol and 0.03% bromophenol blue). The substrate and products were separated using a 12% non-denaturing PAGE and the gel was exposed for autoradiography.

### Immunofluorescence assay

The slides of different intraerythrocytic developmental stages were prepared and after fixing they were blocked in 4% BSA in PBS at 37 °C for 90 min. After washing the slides were incubated with pre-immune serum or anti-PfPSH3C antibodies (raised in rabbit) at 1:500 dilutions and anti-PfDOZI antibodies (raised in mice) at 1:100 dilutions in PBS containing 1% BSA for 2 hrs at 37 °C. After washing the slides were incubated for 1 hr at 37 °C with secondary antibodies; Alexa flour green (Alexa 488) and Alexa flour red (Alexa 594) diluted 1:500 in same solution. Confocal images were collected using a Bio-Rad 2100 laser-scanning microscope attached to a Nikon TE 2000U microscope and the figures were prepared using Nikon NIS Elements software suite version 4 (NIS-Elements AR 4.0.0.0). Pearson’s correlation coefficient (PCC) is the statistical method to simplify fluorescence colocalization analysis. The main advantage of PCC is that it measures pixel to pixel overlap of two images^62^.

### Stage specific western blot analysis of PfPSH3

*P*. *falciparum* 3D7 parasites of both mixed as well as different intraerythrocytic developmental stages (ring, trophozoite and schizont) were harvested from cultures with 10% parasitemia and 4% haematocrit. The lysis was done with 0.15% saponin in RPMI for 20 min on ice followed by PBS wash. The parasite pellets were harvested by centrifugation at 2000 × g for 20 min at 4 °C. The parasite pellets were suspended in lysis buffer (Pierce) containing protease inhibitor cocktail (Roche) and lysed completely using freeze thaw cycles and the supernatants were used for SDS-PAGE analysis. The proteins were transferred overnight to the membrane. After blocking in 3% BSA for 2 hrs, the blots were incubated in pre-immune serum or anti-PfPSH3C antibodies (raised in rabbit, dilution 1:500) and anti-PfH45 antibodies (raised in mice, 1:500) separately followed by detection with HRP-conjugated secondary antibodies (anti-mice or anti-rabbit, Sigma) and incubated at room temperature for 8 hrs. The blots were developed by DAB-peroxide (Diaminobenzidine, Sigma, Saint Louis, MO, USA) staining and the blot was rinsed in 10 mM EDTA to stop the reaction.

### Post translational modifications (PTM) Prediction and STRING analysis

PTMs were predicted using different software for specific PTM such as for Phosphorylation of serine, threonine and tyrosine NetPhos 3.1 (http://www.cbs.dtu.dk/services/NetPhos/)^45^ was used; methylations were predicted by PSSME (http://bioinfo.ncu.edu.cn/PSSMe.aspx)^46^ and acetylation sites were predicted using GPS-PAIL (http://pail.biocuckoo.org/)^47^. Protein-protein interaction prediction was done using STRING-DB Version 10.5 (http://string-db.org/)^48,49^. The cutoff score of 0.7 was used and only top ten interactors are shown in the figure.

## Electronic supplementary material

Supplementary Information
