# Supplementary material for: Plasmodium falciparum specific helicase 3 is nucleocytoplasmic protein and unwinds DNA duplex in 3′ to 5′ direction
Source: Sci Rep. 2017 Oct 13;7:13146. doi: 10.1038/s41598-017-12927-x (PMC5640622; doi:10.1038/s41598-017-12927-x)
Supplement: Supplementary file 1 — Supplementary Information [file 41598_2017_12927_MOESM1_ESM.pdf]

***Plasmodium falciparum* specific helicase 3 is nucleocytoplasmic protein and unwinds DNA duplex in 3' to 5' direction**

Manish Chauhan, Mohammed Tarique and Renu Tuteja*

Parasite Biology Group, International Centre for Genetic Engineering and Biotechnology,
P. O. Box 10504, Aruna Asaf Ali Marg, New Delhi-110067, India
*To whom correspondence should be addressed
Tel. +91-11-26741358; Fax: +91-11-26742316;
Email: renu@icgeb.res.in; renututeja@gmail.com

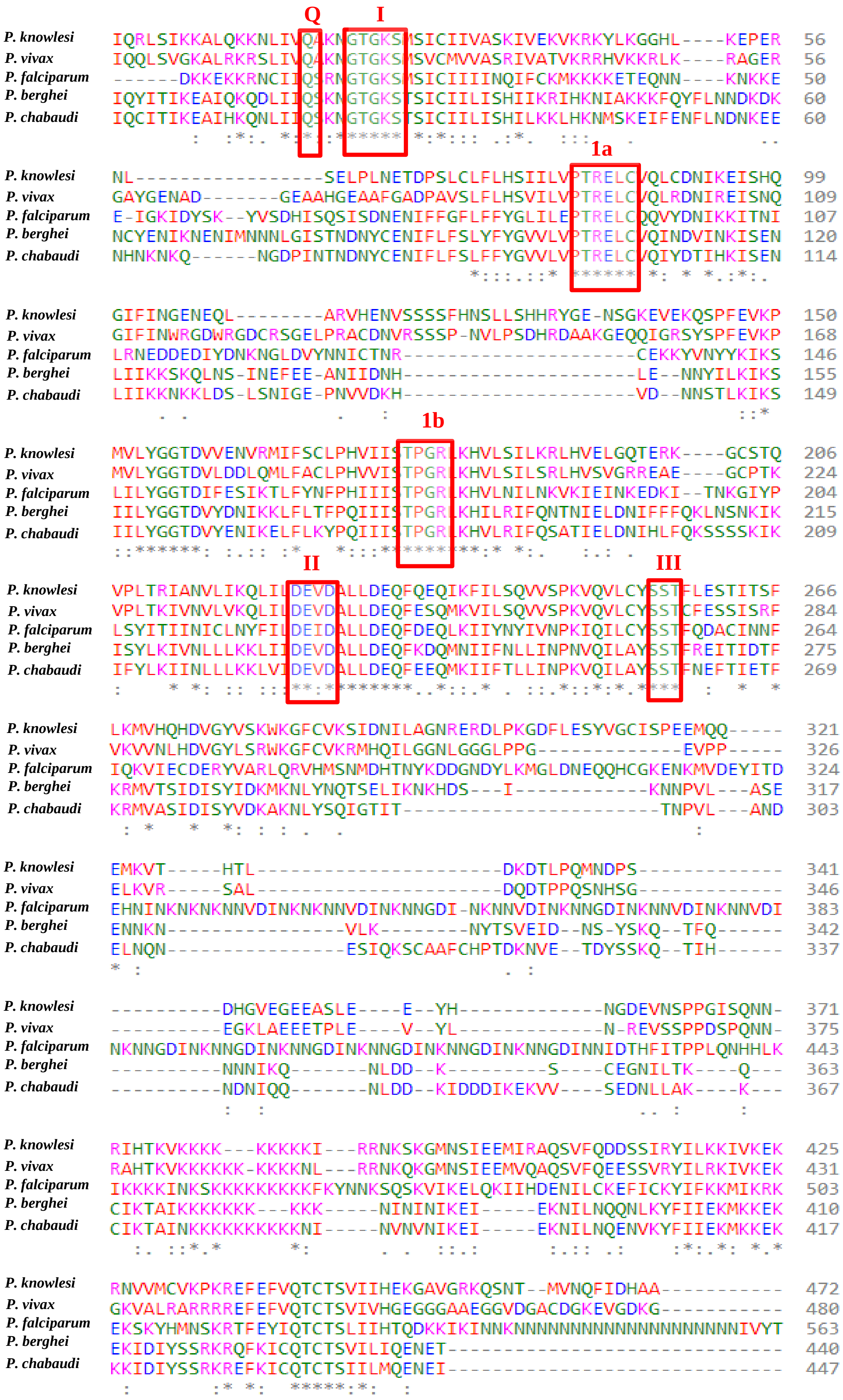

Q
I
1a
1b
II
III
P. knowlesi
P. vivax
P. falciparum
P. berghei
P. chabaudi

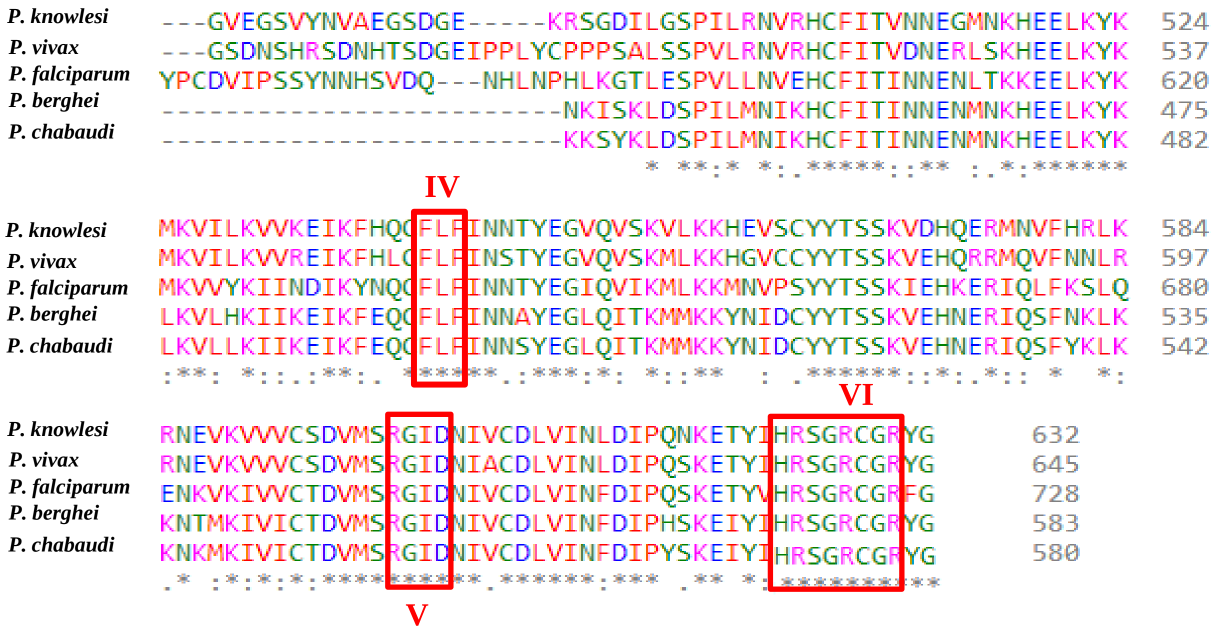

Supplementary Figure 1

Multiple sequence Alignment of the amino acid sequence of core region of PfPSH3 with orthologs present in *Plasmodium* species, *P. knowlesi*, *P. vivax*, *P. berghei* and *P. chabaudi*. The alignment was done using clustal omega program (www.ebi.ac.uk/Tools/msa/clustalo/). All signature motifs of PfPSH3 are highlighted and boxed in red colour and the name of each motif (from I to VI) is written in roman numerals;

A.

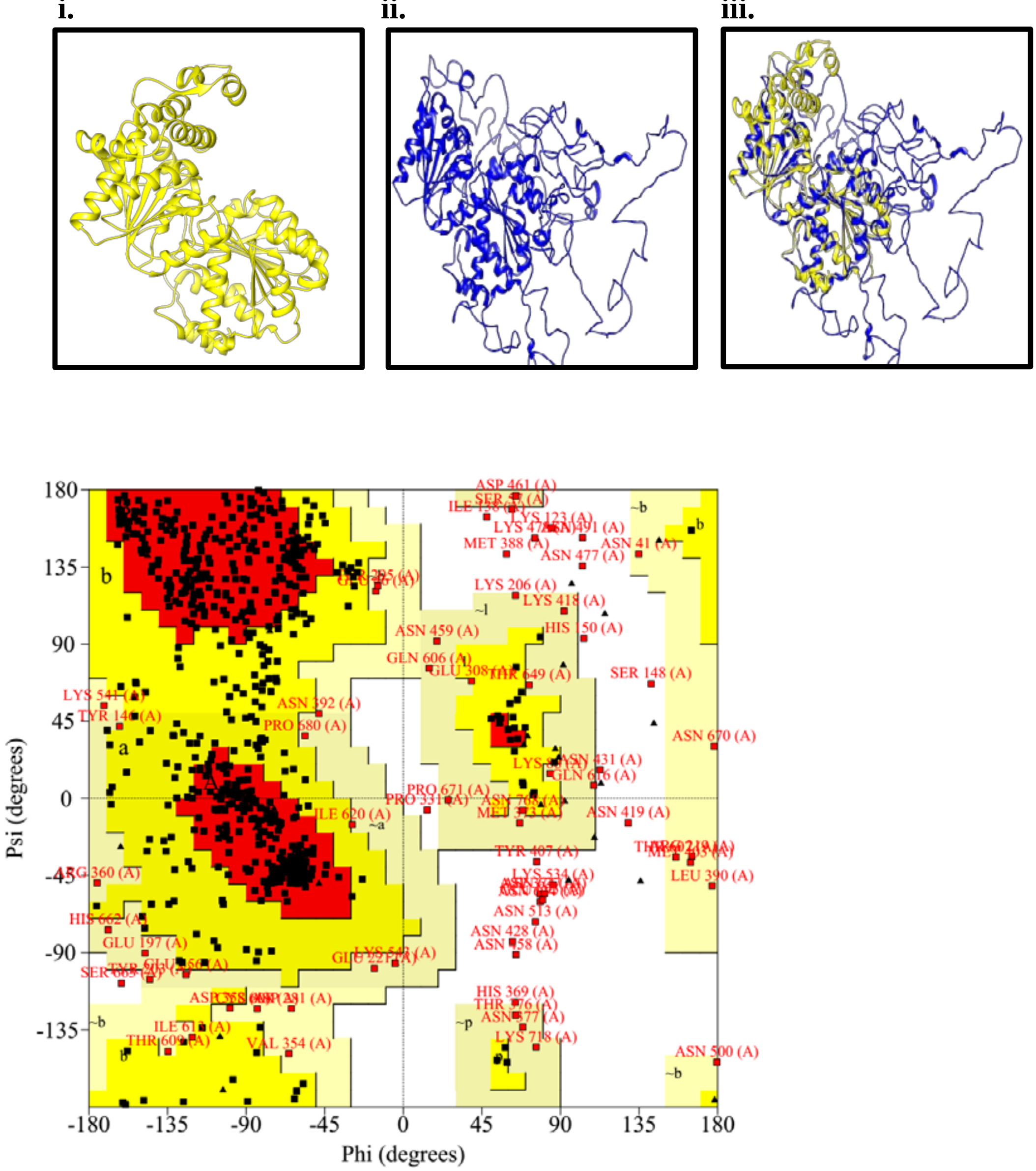

Supplementary Figure 2

(A) Predicted structural model of PfPSH3N and its template 4tyw.1.A (Mss116 mitochondrial helicase, *Saccharomyces cerevisiae*) i. Template; ii. Predicted model of PfPSH3N; iii. Superimposed image of PfPSH3N with its template. All the images were processed using Chimera software; **(B)** Ramachandran plot for the predicted PDB file of PfPSH3N structure was generated using PdbSUM (http://www.ebi.ac.uk/thornton-srv/databases/cgi-bin/pdbsum/GetPage.pl?pdbcode=index.html).

(A) Cloning of PfPSH3

Gene specific primers with appropriate restriction sites were designed based on the available sequences of PfPSH3 at PlasmodB. The primers PfPSH3NF1 (BamHI site at 5′ end GGATCCATGAGTAATGAACCCGTTTCC) and PfPSH3NR1 (with EcoR1 site at 5′ end GAATTCGATTTACAAAAAAAGAACAT) were synthesized to perform the PCR amplification of the 1-3260 base pair N-terminal fragment (PfPSH3N) of PfPSH3. PfPSH3C primers were also synthesized for PCR amplification of the gene fragment from 3420-3966 bp (726 bp) as follows PfPSH3CF1 (BamHI site at 5′ end ATGTTCTTTTTTTGTAAATC) and PfPSH3CR1 (with XhoI site at 5' end TCAACTATCTTCATACAATTTTATATG). The purified PCR products were used for the pJET cloning using CloneJET PCR cloning kit (Thermo Scientific). The sequences have been submitted to the GenBank and the accession numbers for PfPSH3N and PfPSH3C are KY996309 and KY996310, respectively.

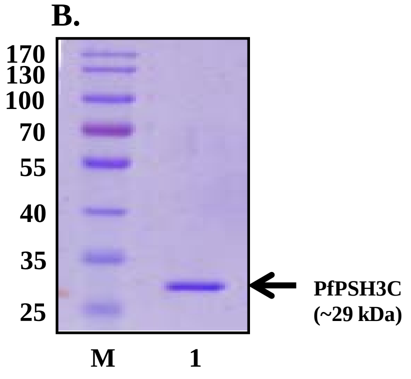

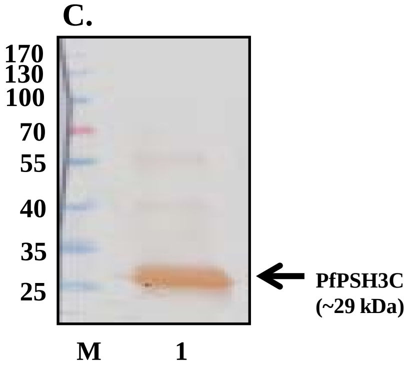

(**B)** Commassie blue stained gel. Lane M is molecular weight marker and lane 1 is purified PfPSH3C protein (~29 kDa); **(C)** Western Blot analysis. Lane numbers are similar to B.

Supplementary Figure 3

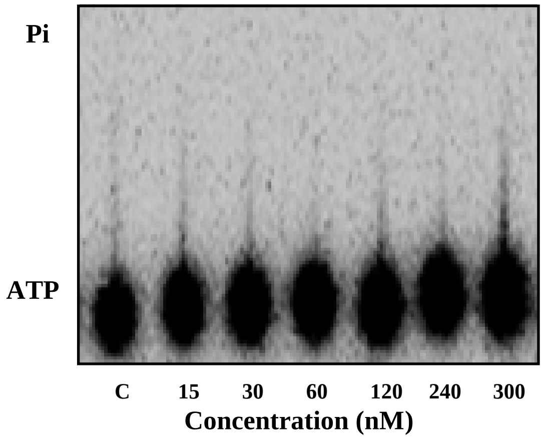

Supplementary Figure 4

ATPase activity in the presence of ssM13 DNA with increasing concentration (15 nM to 300 nM) of PfPSH3C protein (lanes 15–300), lane C is negative control reaction without protein

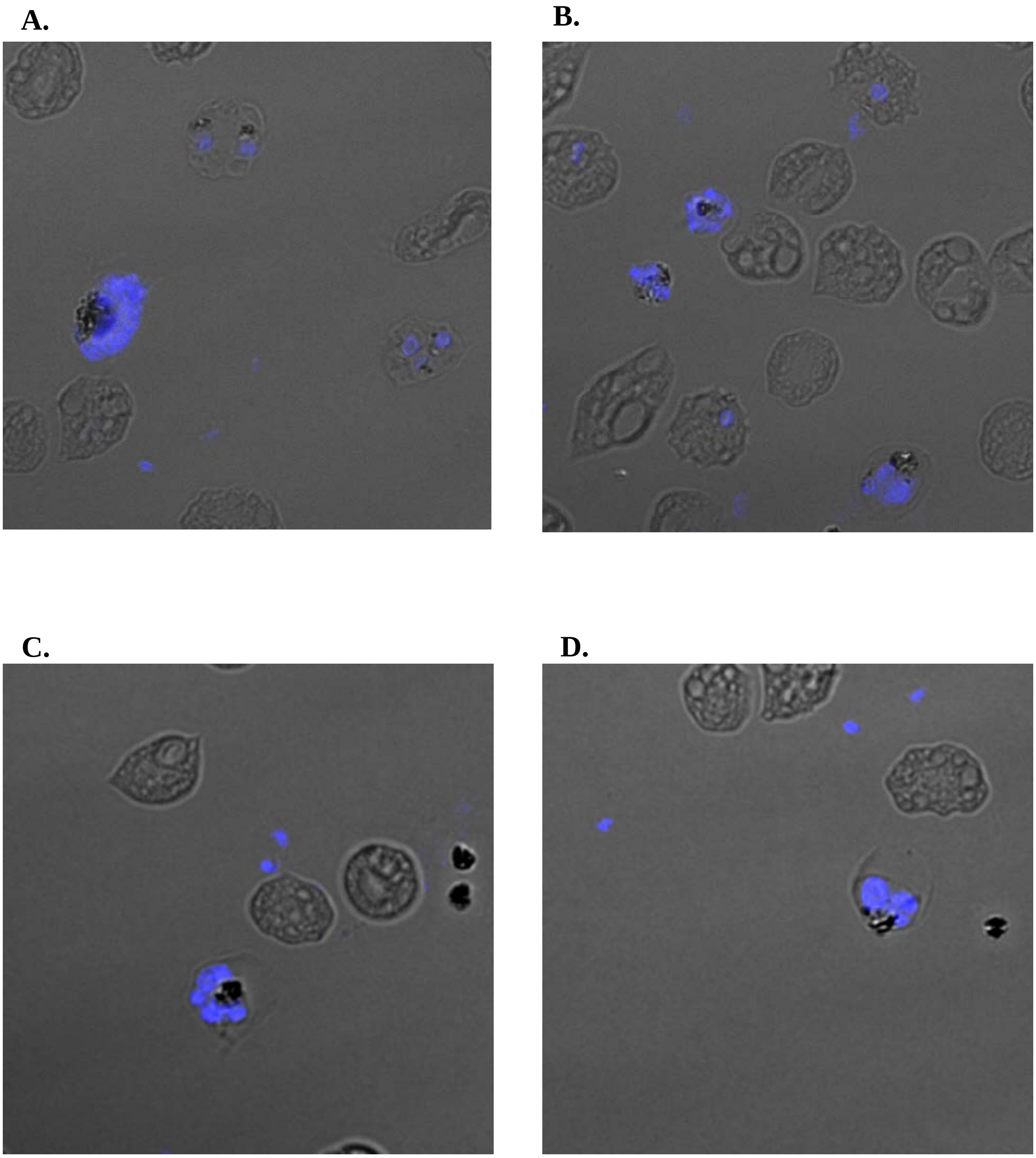

Supplementary Figure 5

Full field view of IFA with preimmune sera at different stages of intraerythrocytic development of parasite. The panels from A to D show parasite pictures taken with combining all the channels (Blue channel for DAPI (nuclear stain) Green channel for preimmune sera and DIC for the morphology of parasite.

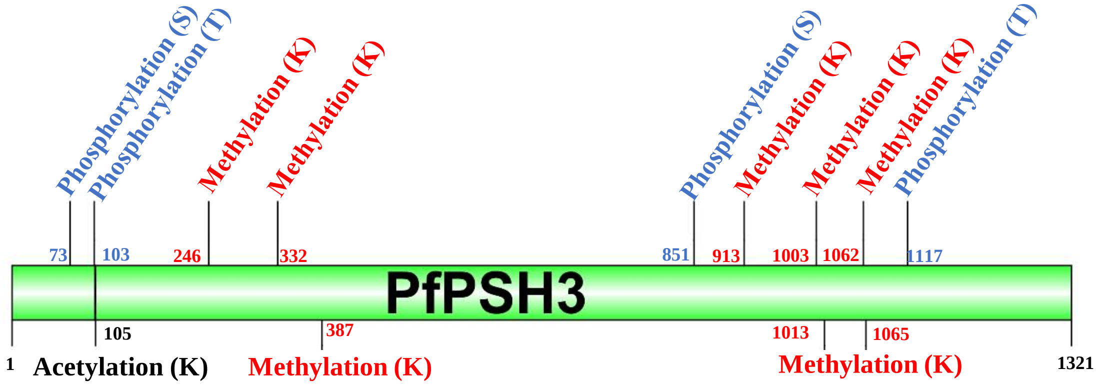

Supplementary Figure 6

Predicted sites for post translational modifications (PTMs) with amino acid number are shown. Phosphorylation at serine and threonine by protein kinase C (PKC) are shown in blue; methylation at lysine residue is shown in red and acetylation is shown in black color.

Supplementary Table 1

A. Phosphorylation prediction Data (www.cbs.dtu.dk/services/NetPhos/)

# Sequence	#	x	Context	Score	Kinase	Answer
# psh3_	2	S	---MSNEPV	0.475	cdc2	.
# psh3_	7	S	NEPVSLISL	0.544	unsp	YES
# psh3_	10	S	VSLISLNKM	0.474	cdc2	.
# psh3_	30	T	NVDNTELTN	0.431	cdc2	.
# psh3_	33	T	NTELTNLVL	0.448	cdc2	.
# psh3_	39	T	LVLNTSNYN	0.654	PKC	YES
# psh3_	40	S	VLNTSNYNK	0.462	CaM-II	.
# psh3_	42	Y	NTSNYNKKE	0.549	EGFR	YES
# psh3_	50	T	EEKGTFEEL	0.565	CKII	YES
# psh3_	57	S	ELNVSIEII	0.655	unsp	YES
# psh3_	70	Y	RINIYYPSV	0.470	INSR	.
# psh3_	71	Y	INIYYPSVI	0.479	INSR	.
# psh3_	73	S	IYYPSVIQY	0.827	PKC	YES
# psh3_	77	Y	SVIQYMIMK	0.570	unsp	YES
# psh3_	99	S	CIIQSRNGT	0.536	PKC	YES
# psh3_	03	T	SRNGTGKSM	0.813	PKC	YES
# psh3_	06	S	GTGKSMSIC	0.521	cdc2	YES
# psh3_	08	S	GKSMSICII	0.447	PKA	.
# psh3_	27	T	KKKETEQNN	0.533	PKC	YES
# psh3_	43	Y	GKIDYSKYV	0.515	INSR	YES
# psh3_	44	S	KIDYSKYVS	0.881	unsp	YES
# psh3_	46	Y	DYSKYVSDH	0.803	unsp	YES
# psh3_	48	S	SKYVSDHIS	0.459	GSK3	.
# psh3_	52	S	SDHISQSIS	0.580	ATM	YES
# psh3_	54	S	HISQSISDN	0.992	unsp	YES
# psh3_	56	S	SQSISDNEN	0.954	unsp	YES
# psh3_	69	Y	FLFFYGLIL	0.429	INSR	.
# psh3_	76	T	ILEPTRELC	0.461	GSK3	.
# psh3_	84	Y	CQQVYDNIK	0.726	unsp	YES
# psh3_	91	T	IKKITNILR	0.476	PKG	.
# psh3_	03	Y	DEDIYDNKN	0.992	unsp	YES
# psh3_	12	Y	GLDVYNNIC	0.507	INSR	YES
# psh3_	17	T	NNICTNRCE	0.738	PKC	YES
# psh3_	24	Y	CEKKYVNYY	0.988	unsp	YES
# psh3_	27	Y	KYVNYYKIK	0.486	INSR	.
# psh3_	28	Y	YVNYYKIKS	0.405	INSR	.
# psh3_	32	S	YKIKSLILY	0.712	PKA	YES
# psh3_	36	Y	SLILYGGTD	0.401	INSR	.
# psh3_	39	T	LYGGTDIFE	0.551	CKII	YES
# psh3_	44	S	DIFESIKTL	0.803	unsp	YES
# psh3_	47	T	ESIKTLFYN	0.467	PKC	.
# psh3_	50	Y	KTLFYNFPH	0.385	EGFR	.
# psh3_	58	S	HIIISTPGR	0.493	PKC	.
# psh3_	59	T	IIISTPGRL	0.679	unsp	YES
# psh3_	84	T	EDKITNKGI	0.515	PKG	YES
# psh3_	289	Y	NKGIYPLSY	0.405	INSR	.
# psh3_	292	S	IYPLSYITI	0.494	cdc2	.
# psh3_	293	Y	YPLSYITII	0.506	INSR	YES
# psh3_	295	T	LSYITIINI	0.443	CaM-II	.
# psh3_	303	Y	ICLNYFILD	0.368	INSR	.
# psh3_	325	Y	LKIIYNYIV	0.464	INSR	.
# psh3_	327	Y	IIYNYIVNP	0.393	INSR	.
# psh3_	338	Y	QILCYSSTF	0.395	INSR	.
# psh3_	339	S	ILCYSSTFQ	0.470	GSK3	.
# psh3_	340	S	LCYSSTFQD	0.448	cdc2	.
# psh3_	341	T	CYSSTFQDA	0.429	GSK3	.
# psh3_	361	Y	CDERYVARL	0.948	unsp	YES
# psh3_	371	S	RVHMSNMDH	0.757	unsp	YES
# psh3_	376	T	NMDHTNYKD	0.439	GSK3	.
# psh3_	378	Y	DHTNYKDDG	0.887	unsp	YES
# psh3_	385	Y	DGNDYLKMG	0.943	unsp	YES
# psh3_	407	Y	MVDEYITDE	0.479	INSR	.
# psh3_	409	T	DEYITDEHN	0.580	CKII	YES
# psh3_	516	T	NNIDTHFIT	0.454	unsp	.
# psh3_	520	T	THFITPPLQ	0.500	GSK3	YES
# psh3_	538	S	KINKSKKKK	0.992	unsp	YES
# psh3_	550	Y	KKFKYNNKS	0.899	unsp	YES
# psh3_	554	S	YNNKSQSKV	0.625	DNAPK	YES
# psh3_	556	S	NKSQSKVIK	0.526	PKC	YES
# psh3_	580	Y	FICKYIFKK	0.485	INSR	.
# psh3_	592	S	RKEKSKYHM	0.688	unsp	YES
# psh3_	594	Y	EKSKYHMNS	0.966	unsp	YES
# psh3_	598	S	YHMNSKRTF	0.834	unsp	YES
# psh3_	601	T	NSKRTFEYI	0.589	PKC	YES
# psh3_	604	Y	RTFEYIQTC	0.731	unsp	YES
# psh3_	607	T	EYIQTCTSL	0.478	p38MAPK	.
# psh3_	609	T	IQTCTSLII	0.526	PKC	YES
# psh3_	610	S	QTCTSLIIH	0.592	PKC	YES
# psh3_	615	T	LIIHTQDKK	0.633	DNAPK	YES
# psh3_	648	Y	NNIVYTYPC	0.454	SRC	.
# psh3_	649	T	NIVYTYPCD	0.481	PKC	.
# psh3_	650	Y	IVYTYPCDV	0.427	INSR	.
# psh3_	657	S	DVIPSSYNN	0.471	GSK3	.
# psh3_	658	S	VIPSSYNNH	0.806	unsp	YES
# psh3_	659	Y	IPSSYNNHS	0.447	INSR	.
# psh3_	663	S	YNNHSVDQN	0.645	unsp	YES
# psh3_	676	T	HLKGTLESP	0.476	GSK3	.
# psh3_	679	S	GTLESPVLL	0.498	p38MAPK	.
# psh3_	691	T	HCFITINNE	0.470	CaM-II	.
# psh3_	698	T	NENLTKKEE	0.960	unsp	YES
# psh3_	705	Y	EELKYKMKV	0.954	unsp	YES
# psh3_	711	Y	MKVVYKIIN	0.617	unsp	YES
# psh3_	719	Y	NDIKYNQCF	0.469	EGFR	.
# psh3_	729	T	FINNTYEGI	0.472	DNAPK	.
# psh3_	730	Y	INNTYEGIQ	0.522	INSR	YES
# psh3_	746	S	MNVPSYYTS	0.472	GSK3	.
# psh3_	747	Y	NVPSYYTSS	0.463	INSR	.
# psh3_	748	Y	VPSYYTSSK	0.469	SRC	.
# psh3_	749	T	PSYYTSSKI	0.457	GSK3	.
# psh3_	750	S	SYYTSSKIE	0.614	PKC	YES
# psh3_	751	S	YYTSSKIEH	0.946	unsp	YES
# psh3_	764	S	QLFKSLQEN	0.943	unsp	YES
# psh3_	776	T	IVVCTDVMS	0.466	GSK3	.
# psh3_	780	S	TDVMSRGID	0.468	GSK3	.
# psh3_	799	S	DIPQSKETY	0.830	unsp	YES
# psh3_	802	T	QSKETYVHR	0.471	PKG	.
# psh3_	803	Y	SKETYVHRS	0.973	unsp	YES

A. continued. Phosphorylation prediction (www.cbs.dtu.dk/services/NetPhos/)

#	psh3_ 807	S	YVHRSGRCG	0.990	unsp	YES
#	psh3_ 821	S	GLCVSLCNY	0.703	PKA	YES
#	psh3_ 825	Y	SLCNYTDYN	0.455	unsp	.
#	psh3_ 826	T	LCNYTDYNY	0.465	cdc2	.
#	psh3_ 828	Y	NYTDYNYLY	0.535	EGFR	YES
#	psh3_ 830	Y	TDYNYLYYF	0.455	INSR	.
#	psh3_ 832	Y	YNYLYYFKY	0.447	INSR	.
#	psh3_ 833	Y	NYLYYFKYK	0.412	INSR	.
#	psh3_ 836	Y	YYFKYKLKL	0.397	INSR	.
#	psh3_ 846	Y	VCDFYYICK	0.494	INSR	.
#	psh3_ 847	Y	CDFYYICKS	0.792	unsp	YES
#	psh3_ 851	S	YICKSDHRK	0.811	PKC	YES
#	psh3_ 859	S	KQDDSSVQI	0.475	cdc2	.
#	psh3_ 860	S	QDDSSVQIN	0.447	PKC	.
#	psh3_ 869	T	NIKKTHEHN	0.436	GSK3	.
#	psh3_ 879	T	QIIDTHSCG	0.475	cdc2	.
#	psh3_ 881	S	IDTHSCGDN	0.977	unsp	YES
#	psh3_ 888	Y	DNNHYKNYD	0.951	unsp	YES
#	psh3_ 891	Y	HYKNYDESG	0.449	INSR	.
#	psh3_ 894	S	NYDESGSSH	0.450	CKII	.
#	psh3_ 896	S	DESGSSHNI	0.548	unsp	YES
#	psh3_ 897	S	ESGSSHNIS	0.446	CaM-II	.
#	psh3_ 901	S	SHNISNFQQ	0.444	GSK3	.
#	psh3_ 908	Y	QQINYNNIF	0.463	INSR	.
#	psh3_ 924	Y	LRKIYENNI	0.445	INSR	.
#	psh3_ 932	Y	IRNDYYEQN	0.512	EGFR	YES
#	psh3_ 933	Y	RNDYYEQNG	0.990	unsp	YES
#	psh3_ 940	S	NGAESLKKK	0.974	unsp	YES
#	psh3_ 959	S	GFVFSKHIN	0.617	PKC	YES
#	psh3_ 968	S	MNQHSYNNT	0.570	unsp	YES
#	psh3_ 969	Y	NQHSYNNTF	0.381	INSR	.
#	psh3_ 972	T	SYNNTFNNV	0.447	CaM-II	.
#	psh3_ 977	Y	FNNVYLKIY	0.630	unsp	YES
#	psh3_ 981	Y	YLKIYFKEL	0.473	INSR	.
#	psh3_ 997	T	QHNNTCSYI	0.446	GSK3	.
#	psh3_ 999	S	NNTCSYIFK	0.580	PKC	YES
#	psh3_1000	Y	NTCSYIFKI	0.473	INSR	.
#	psh3_1022	S	IQHESNLIL	0.463	CaM-II	.
#	psh3_1040	Y	ICCNYYDDH	0.506	INSR	YES
#	psh3_1041	Y	CCNYYDDHI	0.740	unsp	YES
#	psh3_1047	S	DHIKSEHNN	0.918	unsp	YES
#	psh3_1053	S	HNNKSYLYN	0.513	PKC	YES
#	psh3_1054	Y	NNKSYLYNN	0.741	unsp	YES
#	psh3_1056	Y	KSYLYNNTY	0.448	INSR	.
#	psh3_1059	T	LYNNTYFKK	0.619	PKC	YES
#	psh3_1060	Y	YNNTYFKKE	0.488	INSR	.
#	psh3_1066	T	KKEKTKNII	0.440	CaM-II	.
#	psh3_1072	T	NIINTTSFQ	0.583	PKC	YES
#	psh3_1073	T	IINTTSFQT	0.581	PKC	YES
#	psh3_1074	S	INTTSFQTN	0.459	CaM-II	.
#	psh3_1077	T	TSFQTNFCM	0.461	CaM-II	.
#	psh3_1087	S	FFCKSNYYI	0.535	PKA	YES
#	psh3_1089	Y	CKSNYYITL	0.531	unsp	YES
#	psh3_1090	Y	KSNYYITLK	0.626	unsp	YES
#	psh3_1092	T	NYYITLKLY	0.582	PKC	YES
#	psh3_1096	Y	TLKLYYIFL	0.456	INSR	.
#	psh3_1097	Y	LKLYYIFLF	0.413	INSR	.
#	psh3_1105	Y	FLFKYYKYP	0.421	INSR	.
#	psh3_1106	Y	LFKYYKYPL	0.463	INSR	.
#	psh3_1108	Y	KYYKYPLRK	0.402	INSR	.
#	psh3_1115	Y	RKNFYHLLI	0.411	INSR	.
#	psh3_1120	Y	HLLIYNKYY	0.409	EGFR	.
#	psh3_ 1123	Y	IYNKYYNNN	0.511	INSR	YES
#	psh3_ 1124	Y	YNKYYNNNN	0.884	unsp	YES
#	psh3_ 1132	Y	NKEQYYHDE	0.545	unsp	YES
#	psh3_ 1133	Y	KEQYYHDEK	0.942	unsp	YES
#	psh3_ 1143	S	NNNISSFTN	0.431	CaM-II	.
#	psh3_ 1144	S	NNISSFTNL	0.475	CaM-II	.
#	psh3_ 1146	T	ISSFTNLEP	0.513	cdc2	YES
#	psh3_ 1152	T	LEPLTYYLQ	0.458	GSK3	.
#	psh3_ 1153	Y	EPLTYYLQH	0.397	SRC	.
#	psh3_ 1154	Y	PLTYYLQHH	0.371	INSR	.
#	psh3_ 1163	S	NNCISDSYI	0.495	cdc2	.
#	psh3_ 1165	S	CISDSYIYY	0.740	PKC	YES
#	psh3_ 1166	Y	ISDSYIYYK	0.703	unsp	YES
#	psh3_ 1168	Y	DSYIYYKKK	0.515	INSR	YES
#	psh3_ 1169	Y	SYIYYKKKK	0.455	INSR	.
#	psh3_ 1177	T	KKKKTLHKN	0.866	PKC	YES
#	psh3_ 1183	Y	HKNVYNLID	0.527	EGFR	YES
#	psh3_ 1192	Y	EGKLYEQVK	0.860	unsp	YES
#	psh3_ 1197	Y	EQVKYKTNI	0.694	unsp	YES
#	psh3_ 1199	T	VKYKTNINK	0.656	PKC	YES
#	psh3_ 1208	T	HDMFTIHNN	0.641	PKC	YES
#	psh3_ 1213	Y	IHNNYLSNS	0.453	INSR	.
#	psh3_ 1215	S	NNYLSNSIS	0.487	GSK3	.
#	psh3_ 1217	S	YLSNSISYH	0.534	cdc2	YES
#	psh3_ 1219	S	SNSISYHDN	0.968	unsp	YES
#	psh3_ 1220	Y	NSISYHDNL	0.821	unsp	YES
#	psh3_ 1242	S	ININSMSLE	0.599	DNAPK	YES
#	psh3_ 1244	S	INSMSLEQA	0.529	DNAPK	YES
#	psh3_ 1251	S	QAIISLNMD	0.454	DNAPK	.
#	psh3_ 1269	S	LCNESKHIK	0.431	GSK3	.
#	psh3_ 1280	T	NDEITIMKK	0.429	CKI	.
#	psh3_ 1288	Y	KINQYICDQ	0.511	INSR	YES
#	psh3_ 1294	Y	CDQIYINVN	0.981	unsp	YES
#	psh3_ 1300	Y	NVNDYQNVN	0.949	unsp	YES
#	psh3_ 1308	S	NILKSLFLQ	0.552	PKA	YES
#	psh3_ 1318	Y	HIKLYEDS-	0.418	INSR	.
#	psh3_ 1321	S	LYEDS----	0.463	GSK3	.

B. Methylation prediction Data

http://bioinfo.ncu.edu.cn/PSSMe.aspx

Result

Protein name	Position of site	Flanking residues	SVM Probability
3	246	GGTDIFESI-K-TLFYNFPHI	0.92347
3	332	IIYNYIVNP-K-IQILCYSST	0.94508
3	387	YKDDGNDYL-K-MGLDNEQQH	0.94445
3	913	QQINYNNIF-K-NGDIEKLRK	0.96658
3	1003	HNNTCSYIF-K-IPIDNKNIF	0.95854
3	1013	IPIDNKNIF-K-NINIIQHES	0.96311
3	1062	SYLYNNTYF-K-KEKTKNIIN	0.9067
3	1065	YNNTYFKKE-K-TKNIINTTS	0.90095

The first column display the name of the methylation protein. The second column indicates the position of poten methylation site. The third column indicates the arginine(R) or lysine(K) site and its surrounding amino acids orginal sequence(The window length of protein sequence is 19, if sequence not enough, we added vector 'O' represent it.). The fourth column indicates the SVM scores of methylation sites.

C. Acetylation prediction Data

http://pail.biocuckoo.org/

Predicted site(s):

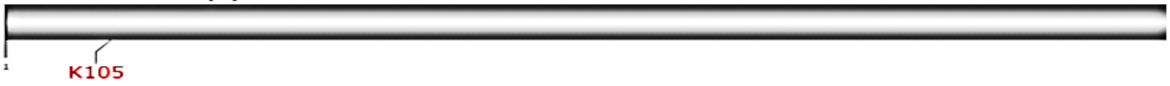

ID	Position	Peptide	HAT	Score	Cutoff
psh3	105	QSRNGTGKSMSICII	HAT1	13.467	9.857

RMgmDB - Rodent Malaria genetically modified Parasites

Rodent **M**alaria **g**enetically **m**odified parasites **D**ata **B**ase (RMgmDB)

http://www.pberghei.eu/index.php?rmgm=3290&hl=PF3D7_0807100

Summary

RMgm-3290

Malaria parasite P. berghei

Genotype

Genetic modification not successful

Disrupted Gene model (rodent): PBANKA_1223500; Gene model (P.falciparum): PF3D7_0807100; Gene product: ATP-dependent RNA helicase DHH1, putative

Phenotype No phenotype has been described

Details of the target gene	
Gene Model of Rodent Parasite	PBANKA_1223500
Gene Model *P. falciparum* ortholog	PF3D7_0807100
Gene product	ATP-dependent RNA helicase DHH1, putative

Details of the genetic modification	
Inducable system used	No
Additional remarks inducable system	
Type of plasmid/construct used	(Linear) plasmid double cross-over

PlasmoGEM (Sanger) construct/vector used	Yes
Name of PlasmoGEM construct/vector	-
Modified PlasmoGEM construct/vector used	No
Plasmid/construct map	
Plasmid/construct sequence	
Restriction sites to linearize plasmid	
Partial or complete disruption of the gene	Complete
Additional remarks partial/complete disruption	
Selectable marker used to select the mutant parasite	hdhfr/yfcu
Promoter of the selectable marker	eef1a
Selection (positive) procedure	pyrimethamine
Selection (negative) procedure	No
Additional remarks genetic modification	The gene is 'likely essential' for asexual blood stage growth/multiplication as determined by barcode PCR in a large pool of gene-deletion mutants.<br>P. berghei blood stages were transfected with a large pool of barcoded disruption (gene-deletion) vectors. These disruption vectors contained long modification arms to efficiently targ the genes of interest. In addition the vectors contain gene-specific molecular barcodes. Co-transfecting multiple gene-deletion vectors in the same electroporation reproducibly generate complex pools of barcoded P. berghei mutants. Unsuccessful gene disruption/deletion or successful gene disruption/deletion is determined by the 'absence or presence' of the barcod in the population (as determined by barcode-specific PCR analysis).<br><br>This report is based on simultaneous phenotyping of mutants by barcode sequencing and is p of a large-scale genetic screen.<br><br>Asexual blood stage phenotype:<br>Growth rate phenotypes were obtained by counting barcodes on a next generation sequence daily between days 4 and 8 post transfection. It is in the nature of the screen that genotypes c individual mutants were not validated.

Supplementary Fig. 7
